# Supplementary material for: Identification of photoperiod-induced specific miRNAs in the adrenal glands of Sunite sheep (Ovis aries)
Source: Front Vet Sci. 2022 Jul 22;9:888207. doi: 10.3389/fvets.2022.888207 (PMC9354845; doi:10.3389/fvets.2022.888207)
Supplement: Supplementary file 1 [file Data_Sheet_1.ZIP › Supplementary file/miRDeep2 file.zip!/miRDeep2 file/Novel_Structure/SPLP42A1/Novel_97.pdf]

Provisional ID	:	NC_019464.2_12859
Score total	:	74.5
Score for star read(s)	:	3.9
Score for read counts	:	66.9
Score for mfe	:	2.1
Score for randfold	:	1.6
Score for cons. seed	:	
Total read count	:	143
Mature read count	:	140
Loop read count	:	0
Star read count	:	3

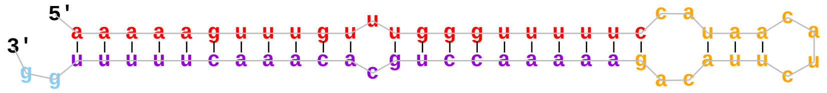

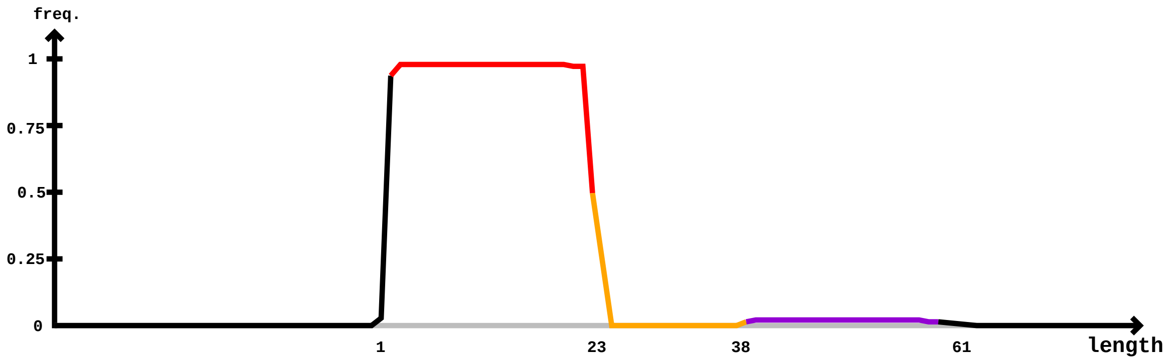

Mature Star

```
5'- ggcucauuaaaaugcuggagcauugaguuggccaaaaaguuuguuuggguuuuuccauaacaucuuacagaaaaaccugcacaaacuuuuuggccagcccaacauguagcc  -3'   obs
    ggcucauuaaaaugcuggagcauugaguuggccaaaaaguuuguuuggguuuuuccauaacaucuuacagaaaaccugcacaaacuuuuuggccagcccaacauguagcc        exp
    ((((((...(((((....)))))(.(((((((((((((((((.((((((((((..(((....)))..)))))))))).))))))))))))))))).)...))).))))   reads   mm   sample
    ................................caaaaaguuuguuuggguuuuu.........................................................   4       0    seq
    .................................aaaaaguuuguuCggguuu...........................................................   1       1    seq
    .................................aaaaaguuCguuuggguuuuu.........................................................   62      1    seq
    .................................aaaaaguuAguuuggguuuuu.........................................................   1       1    seq
    .................................aaaaaguuCguuuggguuuuuc........................................................   66      1    seq
    ..................................Gaaaguuuguuuggguuuuu.........................................................   1       1    seq
    ..................................Gaaaguuuguuuggguuuuuc........................................................   1       1    seq
    ..................................aaaaguuuguuuggguuuuuc........................................................   2       0    seq
    ..................................aaaaguuuguuugAguuuuucc.......................................................   1       1    seq
    ..................................aaaaguuuguuuggguuuuucc.......................................................   1       0    seq
    ......................................................................aaaaaccugAacaaacuuu......................   1       1    seq
    ......................................................................aaaaaccugcacaaacuuuuu....................   1       0    seq
    .......................................................................aaaaccugAacaaacuuuuugg..................   1       1    seq
```
